# Description of a Prospective 17DD Yellow Fever Vaccine Cohort in Recife, Brazil

**DOI:** 10.4269/ajtmh.2011.10-0496

**Published:** 2011-10-01

**Authors:** Andréa Barbosa de Melo, Maria da Paz C. da Silva, Maria Cecília F. Magalhães, Laura Helena Vega Gonzales Gil, Eduardo M. Freese de Carvalho, Ulisses M. Braga-Neto, Giovani Rota Bertani, Ernesto T. A. Marques, Marli Tenório Cordeiro

**Affiliations:** Virology and Experimental Therapy Laboratory, Aggeu Magalhães Research Center, Fiocruz, Recife, Pernambuco, Brazil; Departamento de Bioquímica, Universidade Federal de Pernambuco, Recife, Pernambuco, Brazil; Department of Electrical Engineering, Texas A&M University, College Station, Texas; Center for Vaccine Research, Department of Infectious Diseases and Microbiology, University of Pittsburgh, Pittsburgh, Pennsylvania; Central Laboratory of Public Health, Secretaria de Saúde do Estado de Pernambuco, Recife, Pernambuco, Brazil

## Abstract

From September 2005 to March 2007, 238 individuals being vaccinated for the first time with the yellow fever (YF) -17DD vaccine were enrolled in a cohort established in Recife, Brazil. A prospective study indicated that, after immunization, anti-YF immunoglobulin M (IgM) and anti-YF IgG were present in 70.6% (IgM) and 98.3% (IgG) of the vaccinated subjects. All vaccinees developed protective immunity, which was detected by the plaque reduction neutralization test (PRNT) with a geometric mean titer of 892. Of the 238 individuals, 86.6% had IgG antibodies to dengue virus; however, the presence of anti-dengue IgG did not interfere significantly with the development of anti-YF neutralizing antibodies. In a separate retrospective study of individuals immunized with the 17DD vaccine, the PRNT values at 5 and 10 years post-vaccination remained positive but showed a significant decrease in neutralization titer (25% with PRNT titers < 100 after 5 years and 35% after 10 years).

## Introduction

Yellow fever (YF) is a viral illness transmitted by mosquitoes (*Aedes* and *Haemagogus* genera) infected with the YF virus (YFV), which belongs to the genus *Flavivirus* (family *Flaviviridae*). The clinical manifestations of infection vary considerably, ranging from asymptomatic to classic forms of hemorrhagic fever, which are associated with high fatality rates.^1^

At present, the control of urban YFV transmission can be accomplished by vector control and vaccination. However, YF remains an endemic and epidemic problem affecting millions of people in tropical Africa and South America, especially those living at the fringe of urban and sylvatic environments. It is also a continuing threat to people who travel to these regions without vaccination.^2^ In Brazil, YF is endemic in extensive regions of the Amazon forest and the southwest of the country; therefore, YF vaccination is strongly recommended for travelers to these regions.^3^ The boundary between these two zones is constantly affected by periodic expansions into areas of epizootic activity. YFV is transmitted in sparsely populated forested areas and affects mainly individuals engaged in clearing land for agriculture, ecological tourism, and other professional activities related to penetration of the jungle.^4^ From 1980 to 2008, there were 723 confirmed YF cases of sylvatic origin, among which 389 deaths occurred, resulting in a 53.8% average lethality. Adult males were the most affected segment of the population.^3^

Because of the presence of a large susceptible human population to YFV and the vector *A. aegypti* in the coastal zones of the country, the major urban centers of Brazil are vulnerable to the introduction and spread of YFV. Although there has been no report of urban transmission of YFV in Brazil since 1942, this country is still at risk of YFV outbreaks.^4^

Given the high infestation indices of *A. aegypti* reported in several municipalities of the country and the concomitant high proportion of non-immune humans, there is a high risk of reurbanization of the disease in Brazil. Although *A. albopictus* has been found to be less susceptible to YFV than several *A. aegypti* samples tested from Brazil, *A. albopictus* may continue to colonize non-urban areas of the country, becoming more abundant in rural areas and endemic and transition areas for sylvatic YF at the forest fringes. Thus, this species could become a real problem, because it could become a link between the jungle and urban cycles of YF.^5^–^7^

Two distinct but related lineages (17D-204 and 17DD) of YF vaccine have been developed; these two strains share 99.9% nucleic acid sequence homology. The original 17D strain was developed after 176 culture passages of the wild-type Asibi strain in mouse and chicken tissues. Over 500 million doses have been administered since the vaccine was first developed in 1937. The substrains 17D-204 and 17DD, which are at passages 235–240 and 287–289, respectively, are the ones currently used for the vaccine. The genomes of the Asibi, 17D-204, and 17DD viruses have been sequenced, and the wild-type Asibi differs from the vaccine viruses by 48 nucleotides encoding 20 amino acid substitutions.^8^ The 17D-213 strain, currently at passage 240, is a derivative of 17D-204.^9^,^10^ The 17D-204 vaccine is manufactured in the United States, Switzerland, Russia, Senegal, and China; the 17DD vaccine is manufactured and used in South America.^8^,^11^

The YF vaccine currently used in Brazil is the 17DD strain, which has been produced at the Oswaldo Cruz Institute from an attenuated viral strain.^12^ In 1942, the seed lot system was designed with the goal of reducing the variability of vaccine lot production. In this system, a large lot of virus is produced and extensively verified for titer, sterility, and viral attenuation by assays that include the stringent monkey neurovirulence test. Therefore, vaccine batches produced from this lot are at the same passage level as other lots produced later, providing the necessary production standardization.^13^ Several studies have shown the high efficacy (> 95%) of this vaccine in the prevention and control of the YF epidemic.^14^

In response to the increase in epizootic activity and the threat of urban YFV transmission in recent years, use of the YF vaccine has increased strikingly in Brazil. In addition, a new policy was established in 1998 to include the vaccine in the national program of childhood immunization for people living in an endemic zone. The minimum recommended age for immunization is 9 months. Between 1990 and 2000, about 85 million doses of YF vaccine were administered across the whole country.^15^

The YF vaccine is considered by some vaccinologists to be one of the safest attenuated virus vaccines. Fewer than 25% of vaccinees develop mild systemic symptoms, which may include headache, myalgia, discomfort at the site of vaccination, or low-grade fever from 2 to 6 days after vaccination.^16^ Despite the strong safety profile of the YF vaccines, reports of rare YF vaccine-associated side effects and serious adverse events have been described in the literature, including severe allergic reactions, neurotropic adverse disease, and viscerotropic disease.^16^ In a study published in 2009, YF vaccine-associated viscerotropic disease was estimated to occur in approximately 0.3–0.4 of 100,000 vaccinations, and risk factors identified to date include advanced age and a history of thymus disease. The incidence of YF vaccine-associated neurotropic disease has been estimated to be 0.4–0.8 per 100,000 vaccinations.^17^

The majority of the antibody kinetics studies that have been published are based on data obtained from the 17D strains, and according to Monath,^2^ protective levels of neutralizing antibody are found in 90% of recipients within 10 days and in 99% within 30 days. Immunity is very durable, probably providing lifelong protection after a single dose; to be conservative, revaccination after 10 years is required under International Health Regulations for a valid travel certificate.^2^ In contrast, very few data are available regarding the kinetics of the antibody response to the YF-17DD vaccine used in Brazil. In addition, some reports continue to suggest an increase on the incidence of side effects and deaths related to vaccination, mainly in elderly people and children.^2^,^15^,^18^ The cause of these rare but severe side effects in some individuals is not known.^8^ It is postulated that virus mutations and specific factors in the host's genetic background or concomitant diseases are to blame.^1^,^19^

In addition, dengue virus (DENV) and YFV are related viruses of the *Flavivirus* genus and share cross-reactive antibody epitopes. However, there is still very little understanding on how *Flavivirus* cross-reactive immune responses elicited by natural dengue infections would modulate the immune responses to YF vaccines. It is postulated that the high prevalence of anti-dengue antibodies in the Southeast Asia population is the reason for the absence of YFV in this region. Some studies have also suggested that the existence of anti-dengue immune responses could interfere with the response to the YF vaccine, and it has been shown that previous exposure to DENV in humans may reduce YF viremia, ameliorate the severity of the disease, and reduce the fatality rate without decreasing the rate of infection.^20^ However, there is no evidence suggesting that pre-exposure to a flavivirus could lead to change in the safety profile of YF vaccines.

In this study, we have described the development of a YF cohort of vaccinated individuals living in Recife, the capital of the state of Pernambuco, Brazil, for use in studying the immune responses against the YF-17DD and evaluating possible effects of existing anti-dengue antibodies on the YF vaccine responses. In addition, the samples from this cohort have also been used to map T-cell epitopes elicited by dengue infection and YF-17DD vaccine.

## Materials and Methods

### Cohort design and study population.

**Figure 1.**
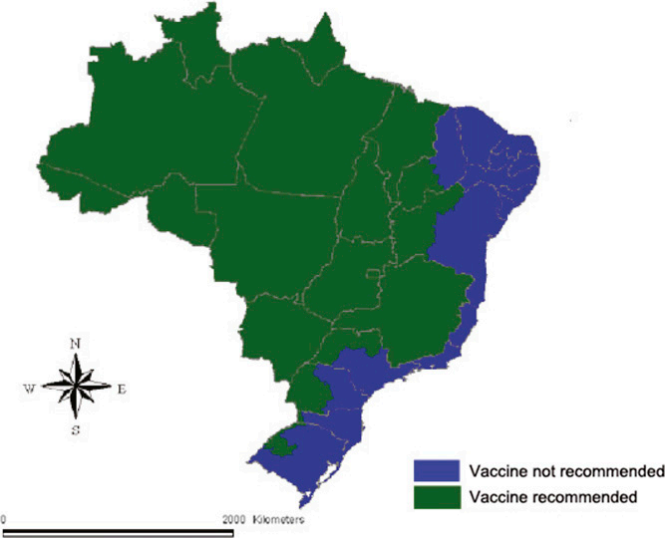
Regions in Brazil where YF vaccination is recommended. Source: SVS/MS, Brazil.

This prospective study was conducted in the city of Recife, Pernambuco, in the northeastern region of Brazil. Recife is located in the coastal non-endemic YF zone, whereas vaccination against YF is recommended only for those traveling to endemic regions (Figure 1). Healthy subjects (*N* = 260) older than 10 years of age who had been immunized for the first time with the YFV-17DD vaccine (Bio-Manguinhos, Oswaldo Cruz Foundation, Rio de Janeiro, Brazil) at the Brazilian National Health Surveillance Agency (ANVISA; located at the International Airport of Recife) were invited to participate in this study. Subjects were enrolled from September 2005 to August 2009. A questionnaire was administered to each subject, and laboratory evaluations were performed before and after vaccination. The subjects were followed with blood sample collection for a period of 4–5 years.

### Ethical considerations.

Written consent was obtained from each subject (or his/her guardian) enrolled in the study. All data were handled confidentially and anonymously. Serological results were shared with each volunteer. This study was reviewed and approved by the Ethics Committee of the Brazilian Ministry of Health (CONEP 12138).

### Blood sample collection.

From each subject, up to six blood samples were collected during the 5-year period of study. The first blood sample was collected before vaccination, and a second sample was collected approximately 30 days later. From then on, blood samples were obtained annually for the remainder of the 5-year study. Sequential blood samples were obtained from 238 of 260 volunteers enrolled in this cohort for a total of 951 samples. Blood samples were collected into 10-mL Vacutainer tubes. Serum samples were separated and stored in cryovials (1 mL per vial) at −20°C for serological analyses. Plasma and peripheral blood mononuclear cells (PBMCs) were also separated and cryopreserved (−80°C) for further studies as previously described.^21^ The cryopreserved samples can serve as a useful tool for future immunological investigations, such as identification of immunodominant human leukocyte antigen (HLA)-restricted T-cell epitopes.

### Laboratory tests.

The first three serum samples collected were tested by enzyme immunoassay, immunoglobulin M (IgM) antibody-enzyme-linked immunosorbent assay (MAC-ELISA), and immunoglobulin G (IgG) antibody-enzyme-linked immunosorbent assay (GAC-ELISA)^22^ and by an indirect IgG-ELISA^23^ to determine levels of IgM and IgG before and after YFV immunization. A plaque reduction neutralization test (PRNT) was used to determine the prevalence of YFV antibodies and/or seroconversion after YF-17DD immunization.^24^ Dengue virus, but no other *Flavivirus*, has been detected in Recife, Pernambuco; thus, all serum samples collected before vaccination were tested for DENV IgG antibodies to study the cross-reactivity with YFV.

### Reference specimens.

Positive and negative control sera used for serological tests were obtained from the Department of Virology—Central Laboratory of Public Health (Secretary of Health of the State of Pernambuco, Brazil). The YF-positive control serum was a pool of 12 serum specimens obtained from individuals who did not have anti-dengue antibody and had been recently vaccinated with YF-17DD. Negative control serum specimens were obtained from individuals who did not have anti-DENV or anti-YFV antibodies.

### YF antigen.

The YF antigen used in the ELISA tests was obtained by the sucrose–acetone method^25^ from the brains of suckling mice infected with YFV (provided by Dr. Pedro Vasconcelos from the Arbovirus Laboratory of the Evandro Chagas Institute, Ministry of Health, Brazil), where it is produced for distribution to Public Health Laboratories in the country.

### YFV.

The human YF vaccine composed of attenuated YF-17DD was obtained from the Oswaldo Cruz Foundation (Rio de Janeiro, Brazil). The vaccine was reconstituted with the distilled water provided, kept in an ice bath, and used for cell inoculation. Vero (African green monkey kidney) cells were grown in minimal essential medium (MEM; Gibco BRL, Gaithersburg, MD) supplemented with 10% fetal bovine serum (FBS; Gibco BRL) and 1% penicillin/streptomycin (Gibco BRL) and kept at 37°C in 5% CO_2_. Subconfluent Vero cells grown in T175 flasks were infected with YF-17DD at a multiplicity of infection (moi) of 0.1 for 5–6 days in MEM with 2% FBS and incubated at 37°C in 5% CO_2_. After freeze-thawing, the cell suspension was centrifuged at 2,000 rpm for 10 min at 4°C. The supernatant containing the YF-17DD particles was collected, it was added to 20% FBS, and it was stored in vials at −80°C. The YFV strain 17DD was propagated in Vero cells at 37°C in 5% CO_2_ to a titer of 1.8 × 10^6^ plaque-forming units (PFU) per mL and used in the PRNT test.

### Anti-DENV IgG antibody detection.

All serum samples collected before YF vaccination and those found to be negative for anti-dengue IgG were tested using the indirect ELISA kit for anti-dengue IgG (PanBio, Pty., Ltd., Brisbane, QLD, Australia). The assay was performed according to the recommended guidelines, and the results were calculated and interpreted as negative or positive according to the manufacturer's instructions.

### Anti-YFV IgM antibody detection.

The protocol reported by Kuno and others^22^ was used for MAC-ELISA with a few modifications. In brief, microtiter plates (Nunc-Immuno Maxisorp, Langenselbold, HE, Germany) were coated with 100 µL of a 1:400 dilution of affinity-purified goat anti-human IgM (Kirkegaard & Perry Laboratories, Inc., Gaithersburg, MD) in carbonate buffer (pH 9.6) and incubated overnight at 4°C. The plates were then washed with phosphate-buffered saline (PBS; pH 7.4) and blotted dry on paper towels between washing steps. After washing, plates were blocked with 200 µL PBS containing 4% bovine serum albumin (BSA) fraction V (Sigma, St. Louis, MO) and incubated for 1 hour at room temperature (RT). After blocking, the plates were washed as before. Patient serum (50 µL) diluted 1:50 in PBS/0.5% BSA was added, in duplicate, to the microtiter well and incubated at 37°C for 1 hour (positive and negative controls sera were included). After washing, 50 µL YF antigen diluted 1:32 in PBS/0.5% BSA (to yield 16 HA units) were added to each microwell and incubated at 37°C for 1 hour. After washing, 25 µL horseradish peroxidase conjugate prepared from an anti-flavivirus monoclonal antibody 6BC1-1 (Centers for Disease Control and Prevention [CDC]) diluted 1:13,000 in PBS/0.5% BSA was added to each well. After another incubation at 37°C for 30 minutes, the plates were washed six times in PBS and then incubated with 100 µL freshly prepared substrate solution 2,2¢-azino-bis (3-ethylbenzthiazoline-6-sulfonic acid) (ABTS; Microwell Peroxidase Substrate System; Kirkegaard & Perry Laboratories, Inc., Gaithersburg, MD) in H_2_0_2_ diluted 1:1. The reaction was allowed to develop in the dark at RT for 30–60 minutes until the positive controls developed a blue-green color. The absorbance was read on a microplate ELISA reader (Bio-Rad Benchmark Plus, Hercules, CA) at 410 nm. After assay validation (absorbance values of control positive serum greater than or equal to five times above values of control negative serum), cut-off values were set at 2.1 times the average absorbance readings for duplicate negative control sera.

### Anti-YFV IgG antibody detection.

The protocol for GAC-ELISA^22^ was nearly the same as that used for MAC-ELISA with a few modifications. Microtiter plates were coated with 100 µL of a 1:200 dilution of affinity-purified goat anti-human IgG (Jackson Immuno Research Laboratories, Inc., West Grove, PA) in carbonate buffer (pH 9.6) and incubated overnight at 4°C before being washed with PBS containing 0.05% Tween 20 (PBS-T; pH 7.4). After washing, the plates were blocked with 200 µL PBS containing 10% non-fat dry milk (NFDM) and then incubated for 1 hour at RT. The plates were again washed in PBS-T as before. Patient serum (50 µL diluted 1:50 in PBS containing 1% NFDM) was added in duplicate to the microtiter wells and incubated at 37°C for 1 hour (positive and negative control sera were included). After washing, 50 µL YF antigen diluted 1:32 in PBS/1% NFDM (16 HA units) were added to each microwell and incubated at 37°C for 1 hour. After washing, 25 µL horseradish peroxidase-conjugated anti-flavivirus monoclonal antibody 6BC1-1 (CDC) diluted 1:13,000 in PBS/1% NFDM were added to each well. After another incubation at 37°C for 30 minutes, the plates were washed six times in PBS-T and then allowed to develop with 100 µL freshly prepared ABTS solution in H_2_0_2_ diluted 1:1. The reaction was allowed to proceed in the dark at RT for 30–60 minutes until the positive controls developed a blue-green color. Absorbance was read on the microplate ELISA reader at 410 nm. After assay validation as described above, cut-off values were set at two times the average absorbance readings for duplicate negative control sera. An index value was calculated by dividing the second sample (after vaccination) absorbance by the first sample (prior vaccination) absorbance for comparing levels of IgG antibodies in both samples. Results were interpreted as follows: < 0.9 (negative); 0.9–1.1 (inconclusive), and > 1.1 (positive; index = sample after vaccine absorbance/sample prior vaccine absorbance).

### Indirect anti-YFV IgG ELISA.

Serum samples were also tested using the protocol adapted from Kuno and others.^23^ In brief, microtiter plates were coated with 100 µL of a 1:32 dilution of YF antigen in carbonate buffer (pH 9.6) and incubated overnight at 4°C. Before use, the plates were washed four times with PBS-T, blocked with 200 µL PBS/10% NFDM, and then, incubated for 1 hour at RT. After washing, 50 µL patient sera diluted 1:50 in PBS/1% NFDM were added in duplicate to the plate wells and incubated at 37°C for 1 hour (positive and negative control sera were also tested). After washing, 25 µL affinity-purified goat anti-human IgG conjugated with horseradish peroxidase (Zymed Laboratories Inc., San Francisco, CA) diluted 1:1,000 in PBS/1% NFDM were added to each well, and the plates were incubated at 37°C for 1 hour. The plates were then washed six times in PBS-T and incubated with 100 µL freshly prepared tetramethylbenzidine (TMB; BD Biosciences Pharmingen, San Diego, CA) substrate solution in the dark at RT for 30–60 minutes until the positive controls developed color. The absorbance was read on a microplate ELISA reader at 450 nm (630-nm reference filter). After assay validation as described above, cut-off values were set at 2.1, which was two times the average absorbance readings of duplicate negative control sera after subtraction of background readings from a blank control well.

### PRNT.

The PRNT was performed using a modified protocol of Morens and others.^24^ Samples collected from the same volunteer were processed together in the same set of tests. Tests were carried out on Vero cells that had been seeded at a density of 300,000 cells/mL and grown in MEM/10% FBS in 24-well microplates (0.5 mL/well) for 24 hour before assay. Human serum samples from YF-17DD vaccinees were inactivated for 30 minutes at 56°C before being diluted in MEM. The assay for the detection of YFV neutralizing antibodies was performed after serial twofold dilution of serum (1:20 to 1:2,560) into 96-well microtiter plates and addition of 30 PFU of challenge virus (17DD YFV strain) to all wells. After incubation at 37°C in a 5% CO_2_ atmosphere for 1 hour, the medium of the 24-well microplates was discarded, and 50 µL each dilution of the mixture serum/virus were inoculated in triplicate. The plates were then incubated at 37°C in 5% CO_2_ for 1 hour to allow virus adsorption. After incubation, the cells were overlaid with 0.5 mL MEM medium containing 10% FBS, 1% penicillin/streptomycin, and 2.7% carboxymethylcellulose. After incubation for 6–7 days at 37°C in 5% CO_2_, the cell monolayer was fixed with formalin and stained with crystal violet, and the plaques were counted. Standard sera of known antibody content were included in each set of tests (negative < 1:20 and positive = 1:320). The 50% endpoint dilution of each serum, corresponding to the dilution at which 50% of the wells were completely protected from infection, was determined according to the Reed–Muench method.^26^ Plaque neutralization titers were calculated as the highest dilution of antibody reducing 50% of the plaques of input virus. All PRNT assays were completed by the same technician using the same standard assay procedure.

### Seroconversion.

To document YF-17DD seroconversion, the antibody profile obtained at the beginning of the study (before vaccination) was compared with the antibody status determined in subsequent serum samples. First, second, and third serum samples collected from volunteers were analyzed by PRNT; first and second samples were analyzed by ELISA anti-YFV IgM, anti-YFV IgG, and anti-dengue IgG.

### Database.

The study data were integrated into a customized digital database that included a questionnaire, serological and research results, and the respective inventories of cryopreserved samples. This FileMaker-based database has been programmed and hosted by the Department of Virology, Centro de Pesquisas Aggeu Magalhães (CPqAM)-Fiocruz and has been used for cohort management and patient/sample tracking.^27^

### Retrospective study.

We also performed a retrospective analysis of a random sample of individuals who had been vaccinated 5 (*N* = 20) or 10 (*N* = 20) years ago with the 17DD vaccine. These individuals were randomly selected from the ANVISA YF vaccine database, contacted by telephone, and invited to participate of the study in 2007. The objective was to investigate the levels of YF neutralizing antibodies at 5 and 10 years after immunization. A single blood sample was collected from each participant for analysis of YF antibody titers by PRNT as described above; the capture anti-YFV IgG ELISA was also performed in these samples. Statistical analyses were conducted as described below.

### Statistical analysis.

Statistical analyses were performed with GraphPad Prism version 4.0 for Mac (GraphPad Software, Inc.) and the R program (R Foundation for Statistical Computing). Antibody titers after vaccination were analyzed using the general linear model Y_ijk_ = μ + P_i_ + D_j_ + V_k +_ e_ijk_, where Y is the response variable, μ is the general average of the experiment, P is the individual effect of collaborators, D is the presence of antibody against DENV before and after YF vaccination, V is the effect of the period between YF vaccination and moment of YF antibody titer evaluation included as a covariate, and e_ijk_ is the residual effect normal independent distribution (NID; 0, σ^2^; average 0 and constant variance). The time between vaccination (5 and 10 years) and serum neutralization test in the retrospective study was analyzed using the general linear model Yi = m + Ti + e_i_, where Y is the response variable, m is the general average of the experiment, and T is the time between vaccination and serum neutralization test. A non-parametric Wilcoxon test was used to compare the YF IgG and YF PRNT indices between positive and negative dengue patients. Non-linear regression of the YF IgG and YF PRNT indices as a function of time post-vaccination in days was performed by a locally weighted polynomial regression (lowess) implemented by the R function plot.smooth.line.

## Results

### Volunteer recruitment.

The recruitment of vaccination candidates for the prospective study began in September 2005. The volunteers consisted of healthy YFV vaccine-naïve individuals living in Recife, Brazil, who were 10 years of age or older and were traveling to regions of Brazil and/or abroad where YF vaccination is recommended. Recruitment ended in March 2007.

### Study cohort characteristics.

From September 2005 to August 2009, a total of 951 blood samples from 260 YF-17DD vaccinated volunteers was obtained and assayed. Serum, plasma, and PBMC samples were stored. A total of 3,200 PBMC sample vials (4 × 10^6^ or 8 × 10^6^ cells per vial) was cryopreserved for further studies. Of the 260 initial cohort volunteers, 22 (8.5%) were excluded from the study, because it was not possible obtain follow-up samples. Thus, the final cohort consisted of 238 subjects who each provided a set of three to six consecutive samples, with an average of four blood samples per subject. In general, the vaccine was well-tolerated, and there were no reports of severe adverse events. Only three participants (29, 34, and 57 years old) reported fever, and one subject (43 years old) reported pain in the injection site. The age range of the volunteers was 14–79 years (median age = 32 years); 86 (36.1%) were female, and 152 (63.9%) were male. There was only one volunteer younger than 15 years and six volunteers older than 70 years; the majority of the volunteers were older than 17 years of age.

### Baseline sampling: anti-YFV IgG and anti-DENV IgG.

To confirm that the volunteers were being immunized for the first time, all samples collected before immunization were tested for anti-YFV IgG antibodies using both IgG ELISA techniques, and none of the 260 serum samples obtained before vaccination revealed any anti-YFV antibody. To determine whether previous exposure to dengue had a significant effect on the YFV vaccination results, we investigated the presence of anti-dengue IgG in this cohort. The serological tests performed on the pre-vaccination samples revealed that 82% (195/238) of the volunteers were positive for anti-dengue antibodies. Interestingly, among the 43 anti-dengue negative subjects, 21% (9/43) presented anti-dengue IgG in the next blood sample, which was taken approximately 1 month after immunization. Two other anti-dengue IgG-negative individuals seroconverted later, one 2 months later and another subject 1 year later. None of these individuals reported having had dengue fever; nevertheless, it is known that asymptomatic dengue cases are common, and in this period, dengue epidemics have occurred in this region. It is not clear if this seroconversion was caused by dengue infection or cross-reaction between anti-YFV antibodies and the DENV epitopes. At the end of the study 4 years later, 13.4% of the volunteers (32/238) remained negative for DENV. In summary, 86.6% (206/238) of subjects were considered dengue IgG-positive, and in 13.4% (32/238) of the subjects, anti-dengue IgG was not detected.

### Evaluation by ELISA of antibody responses to YF-17DD vaccination.

**Figure 2.**
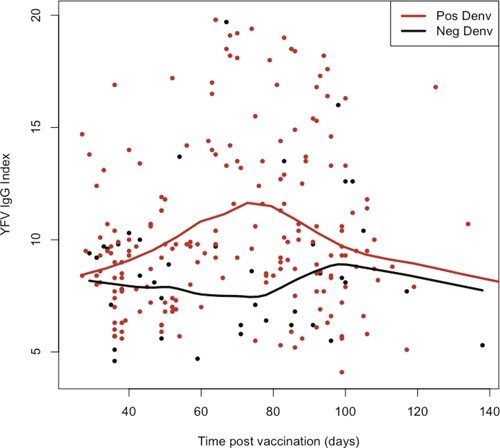
Comparison of YFV IgG index between anti-dengue–positive and -negative individuals after YF-17DD vaccination (*N* = 238).

YFV IgM and IgG antibody detection was performed on 498 first and second serum samples. Of the 238 serum samples obtained after vaccination, anti-YFV IgM was detected in 70.6% (168/238). Among the 70 anti-YFV IgM-negative subjects, 18.6% (13/70) and 81.4% (57/70) of subjects were negative between days 27 < 60 and 60 < 348 for anti-YFV IgM, respectively (Table 1). It was observed that 91.5% (64/70) of the anti-YFV IgM-negative volunteers had previously been exposed to DENV, whereas 83.4% (140/168) of the anti-YFV IgM-positive subjects had been exposed to dengue; however, this difference was not statistically significant. Anti-YFV IgG assays, both capture and indirect ELISA, showed that 98.3% (234/238) of subjects were positive for IgG, whereas ~1.7% (4/238) remained negative (Table 1). Three of four seronegative volunteers were also YFV-IgM–negative (on days 100, 106, and 348). Despite all four YFV IgG-negative subjects having detectable anti-dengue IgG, the YFV IgG index was higher in all points of the curve in anti-dengue IgG-positive than anti-dengue negative individuals (Figure 2). The difference was statistically significant with *P* < 0.001.

Female volunteers had a lower seroconversion rate for IgM than did males (64% versus 74%). However, this difference was not statistically significant.

### Evaluation by PRNT of neutralizing antibody responses to YF-17DD vaccination.

For the analysis of the protective immune response after vaccination, the PRNT is currently considered the gold standard.^28^ PRNT measures antibodies against neutralizing epitopes, whereas ELISA measures binding antibody against non-neutralizing and neutralizing epitopes. The main difference is that PRNT determines antibody functionality and is correlated with protective immunity. This assay is more sensitive and the most specific of the serologic tests.^29^,^30^ Therefore, in addition to the IgM and IgG ELISAs, we also performed PRNT on samples collected before and after vaccination.

**Figure 3.**
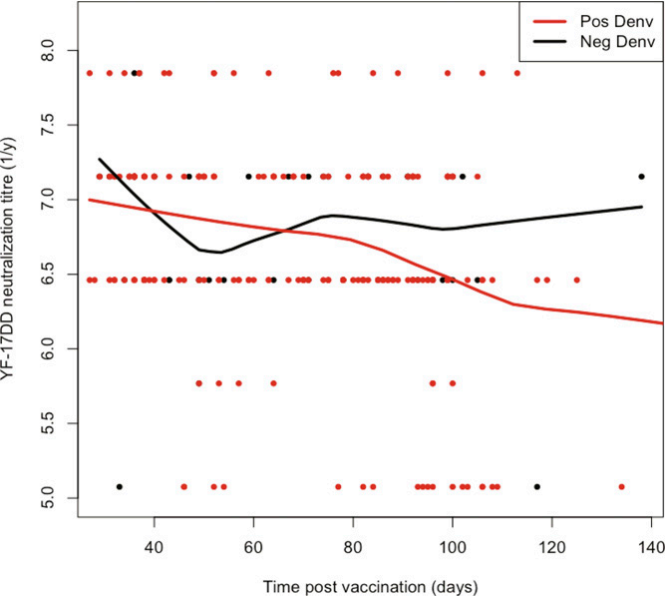
Comparison of neutralization antibody titers between anti-dengue–positive and -negative individuals after YF-17DD vaccination (*N* = 238).

None of the samples collected before vaccination showed neutralizing antibodies to YFV, including those samples with anti-DENV antibodies. Analysis of the prospective cohort showed that 100% of the individuals developed a protective humoral immune response, which was defined as a PRNT titer of ≥ 1:20, including the three individuals who did not produce detectable amounts of anti-YFV IgG or IgM. Post-vaccination PRNT values fluctuated during the first 3 months. The higher neutralization titer found had a geometric mean titer (GMT) of 1,810 (95% confidence interval [CI] = 744–3096), and the lowest neutralizing titer found had a GMT of 452 (95% CI = 160–640). A more detailed analysis of the PRNT results is shown in Table 2. In the 238 serum samples analyzed, those from subjects without antibodies to DENV had a higher neutralizing titer against YFV (GMT = 884) than those with anti-DENV IgG antibodies (GMT = 781). However, Figure 3 shows that this difference was not significant.

### Retrospective study.

**Figure 4.**
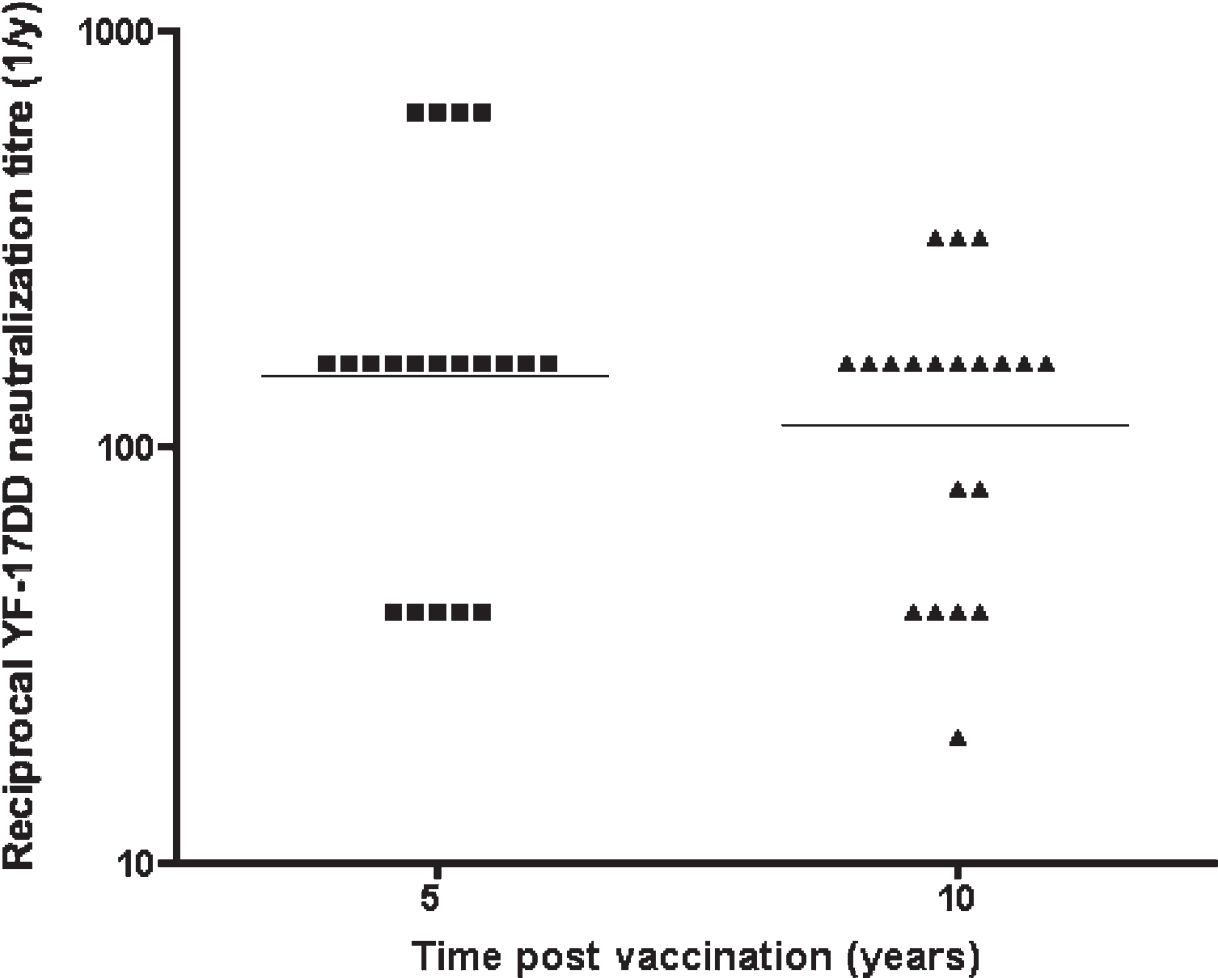
Neutralization antibody titers 5 and 10 years after YF-17DD vaccination (*N* = 40).

Individuals who had been immunized 5 or 10 years earlier for YFV were randomly selected from the ANVISA immunization records. The 40 individuals who agreed to participate had received YF-17DD either 5 (*N* = 20) or 10 years ago (*N* = 20). The age of the volunteers ranged from 16 to 59 years (median age = 42.5 years; standard deviation [SD] = 11.5) for the 5-year group and from 30 to 83 years (median age = 50.5; SD = 15.4) for the 10-year group. Of the 40 volunteers, 28 (70%) were female; the overall age range was 16–83 years (median = 46 years). These samples were submitted to the capture anti-YFV IgG ELISA; 60% (12/20) and 55% (11/20) of the individuals immunized were anti-YFV IgG-positive 5 or 10 years post-vaccination, respectively. However, neutralizing antibodies against YFV were detected in all 40 subjects. In the 5-year post-vaccination group, the PRNT titers varied from 40 to 640, with a GMT of 149 (95% CI = 124–328), and in the 10-year post-vaccination group, they showed a further decrease in titer, with a GMT of 113 (95% CI = 102–188) and a range of titers from 20 to 320 (Figure 4). The anti-YFV IgG-negative samples were found mainly in those individuals presenting PRNT titers from 1:20 to 1:80; 25% (5/20) of the individuals had PRNT titers below 100 at 5 years post-immunization, whereas 35% (7/20) had presented a titer below 100 at 10 years after immunization. This difference was not significant (data not shown). The ELISA results have shown that PRNT is a more sensitive assay, and they emphasize the importance of using this test when measuring protective immunity.

## Discussion

In the present study, we have established and analyzed the immunologic responses of a cohort of individuals living in Recife, Brazil, that had been vaccinated against YF with the 17DD vaccine. It is known that both the 17D and 17DD YFV vaccines induce long-lasting immunity in nearly 100% of individuals vaccinated with a single dose.^2^ The immunological profile of the 17DD vaccine has always been assumed to be identical to that of the 17D vaccine; however, a formal demonstration of the immunological profile of 17DD has been lacking in the scientific literature. In addition, it is not known if previous infection by dengue would interfere with the 17DD vaccine response. In our prospective study, all volunteers had neutralizing antibodies at 1 month after vaccination (GMT = 912); in the retrospective study, the same was true at 10 years post-vaccination (GMT = 113), although at much lower titers. These results are similar to those previously obtained with the 17D vaccine^31^ and suggest that a single dose of the YF-17DD vaccine may provide protection from wild-type YFV infection for at least 10 years, which is consistent with the World Health Organization (WHO) recommendation for international travel to YF-endemic areas. The minimum PRNT titer necessary guarantee protection is not clear, and because 35% of the individuals presented titers below 1:100 after 10 years post-vaccination, the WHO recommendation to repeat YFV immunization every 10 years is very reasonable.^29^,^32^

The YF vaccine is regarded as one of the safest attenuated virus vaccines with few adverse reactions; nevertheless, fatal cases associated with YF-17DD vaccine in Brazil were reported during a period of intensified vaccination in the country.^15^ Most of these adverse reaction cases were in elderly and immune-compromised individuals. Indeed, a study from the United States reported higher frequency of severe adverse events in elderly people, with those older than 75 years having a risk 12 times higher than that of young adults.^18^ It was not the intent of this study to determine the frequency of 17DD adverse reactions given the small sample size of our YF cohort; however, four subjects (1.7%) reported fever or discomfort at the injection site. The precise frequency of severe adverse effects remains uncertain; according to several studies, it may be approximately 1 in 10,000, with a lethality rate of less than 1 in 100,000.^2^ In contrast to other live attenuated vaccines, the 17DD vaccine is quite safe. The neurologic disorders after measles–mumps–rubella and oral poliovirus vaccines are reported at the rate of 1 case per 2.5 million^33^ and 0.06 per 100,000^34^ vaccinees, respectively. Despite the fact that the YF vaccines are considered effective and very safe, their use is not recommended in some circumstances, such as breastfeeding, immunological diseases, and allergies to egg products among others, and it is important to develop a new YF vaccine using new production strategies to have less adverse events.

Enzyme immunoassay has become the method of choice for rapid serodiagnosis of virus infections by detection of specific IgM antibodies and has been frequently applied in a quantitative assay format to monitor immune response to vaccines.^35^ Serologic diagnosis is generally accomplished by measurement of IgM antibodies by ELISA, hemagglutination inhibition (HI), and neutralization.^2^ Here, we have analyzed the humoral immune response against YFV after vaccination over long and short periods of time. In the 238 individuals whose serum samples were analyzed by MAC-ELISA, anti-YFV IgM was detected in 70.6% of cases. Studies have shown that a significant amount of IgM antibody can be present as long as 60^30^ and 82 days^36^ after primary vaccination against YF-17D. In some individuals, anti-YFV IgM can persist for 100–120 days or more.^2^,^37^ Here, we observed that anti-YFV IgM was detected in 15 samples collected 100 days after vaccination. In a study of 17 naïve individuals vaccinated with YF-17D reported by Vazquez and others,^35^ it was found that 94% seroconverted, and no cross-reaction with dengue antigen was observed using the MAC-ELISA test. In another study, Lhuillier and others^37^ found high specificity of IgM antibodies response in individuals with YF infections. The lack of detection of IgM in ~30% of the vaccinees enrolled in this study is most likely because of the time of the blood collection, which was typically more than 30 days post-vaccination. However, it is known that, in secondary flavivirus infections, the level of IgM is lower than in primary infection, and in many cases, it can be undetectable.^38^ However, it could be also considered as a false negative because of the low sensitivity of MAC-ELISA to detect IgM. The YFV-IgG seroconversion was detected in 98.3% (234/238) of the cohort volunteers, and the four vaccinees that did not seroconvert were anti-dengue IgG-positive. Nevertheless, these four individuals showed significant PRNTs.

YFV and DENV share epitopes and therefore, induce cross-reactive antibodies, which often cause difficulties in differentially diagnosing flavivirus infections.^39^ There is a clear chance of obtaining false-positive results because of antibodies cross-reacting with similar epitopes found in other flaviviruses (resulting from either natural infection or vaccination).^28^

In Brazil, several arboviruses (Mayaro, Oropouche, YF) have been responsible for outbreaks of acute febrile illness in the Amazon region and on the Central Plateau.^4^ In addition, one report has also suggested presence of Saint Louis Encephalitis virus. However, the only arbovírus detected in the northeastern region of Brazil for the past 30 years was DENV. For the past 25 years, dengue fever has been considered a serious public health problem in Brazil. The northeastern region of Brazil is one of the most severely affected areas in the country,^21^ and in the case of dengue, Pernambuco is the second most-affected state in this region, with an incidence rate ranging from 134 to 1,438 per 100,000 inhabitants (in 1995 and 2002, respectively). In Recife, the capital of Pernambuco, the dengue incidence rate ranged from 28 to 2,378 per 100,000 inhabitants in the same period.^38^ From 2003 to 2010, Recife experienced a low endemic period, with an average of 37 cases per 100,000 inhabitants, despite the presence of three DENV serotypes (DENV-1, -2, and -3). This decline was probably caused by either an intensification in the vector control measures or a high prevalence of dengue immunity in the population.^40^ A population-based household dengue survey carried out in three distinct areas of the city of Recife between 2005 and 2006 showed a high dengue seroprevalence in the region, with prevalences of 91.1%, 87.4%, and 74.3%, respectively, in deprived, intermediate, and high socio-economic areas.^41^ Thus, the presence of dengue IgG antibodies in 86.6% of subjects enrolled in the YF cohort is consistent with these data.

ELISA assays for YF and DENV had a high cross-reactivity, resulting in rises of IgG titers in secondary infections.^39^ It was found that the YFV IgG was higher in volunteers that had previously been exposed to DENV. Reports presented by other researchers show a higher cross-reactivity between these flavivirus.^28^,^30^ Because PRNT has been proposed as the assay of choice for differentiating YFV and DENV infections,^39^ all serum samples were also subjected to the neutralization test. Despite that the other studies have shown that DENV-naive and DENV-exposed subjects developed different neutralizing titers against YFV,^42^,^43^ this profile was not found in our analysis.

Gender differences in vaccine immunogenicity have been found in other studies^44^,^45^ and were considered in the present study. Anti-YFV IgM antibodies detected by ELISA showed a gender-specific immune response; nevertheless, slightly higher neutralizing titers in the PRNT were found in males, but this difference was not statistically significant. The reason for stronger immune response in men than in women after YF vaccination remains unclear, although this phenomenon has been reported before.^28^

In conclusion, our analysis of the immune response to YF immunization with 17DD yielded an immunological profile identical to that of the 17D vaccine. 17DD was showed to be highly immunogenic, producing 100% seroconversion by PRNT and long-term persistence of neutralizing YFV antibodies 10 years after vaccination. Additionally, the immunogenicity does not seem to be significantly affected by previous exposure to natural dengue infection.

## Figures and Tables

**Table 1:** Detection of YFV-specific IgM and IgG antibodies according to the time of sample collection (*N* = 238)

Number of days since immunization	IgM antibodies		IgG antibodies	
	Negative (%)	Positive (%)	Negative (%)	Positive (%)
27–40	6 (12.8)	41 (87.2)	0 (0)	47 (100)
40–60	7 (14)	43 (86)	1 (2)	49 (98)
60–80	15 (35.7)	27 (64.3)	0 (0)	42 (100)
80–100	25 (36.2)	44 (63.8)	0 (0)	69 (100)
100–349	17 (56.7)	13 (43.3)	3 (10)	27 (90)
Total	70 (29.4)	168 (70.6)	4 (1.7)	234 (98.3)

**Table 2:** Follow-up of neutralization titer after YF-17DD vaccination (2005–2009)

Months post-vaccination	Number of samples (serum)	Geometric mean titer	95% CI	Median PRNT titer of reactive sera	PRNT titer (minimum to maximum)
1–2	94	912	966–1,230	1,280	160–2,560
2–3	75	829	848–1,087	640	80–2,560
3–4	62	626	677–985	640	160–2,560
4–7	9	941	576–1,877	1,280	160–2,560
7–8	4	1,810	744–3,096	1,920	1,280–2,560
8–9	7	1,280	721–2,205	1,280	640–2,560
9–10	8	1,280	811–2,309	1,280	320–2,560
10–11	32	1,174	1,138–1,712	1,280	160–2,560
11–12	31	1,024	971–1,517	1,280	160–2,560
12–13	41	1,045	1036–1,540	1,280	160–2,560
13–14	24	1,046	941–1,526	1,280	160–2,560
14–15	12	678	451–1,523	640	160–2,560
15–16	4	761	291–1,309	640	640–1,280
16–17	4	452	139–902	640	160–640
